# A Novel Silicone Simulation Model for Microvascular Anastomosis

**DOI:** 10.1177/22925503211003835

**Published:** 2021-05-06

**Authors:** Jessica M. Winter, Christian Petropolis

**Affiliations:** 1 8664 University of Manitoba, Winnipeg, Manitoba, Canada

**Keywords:** surgical simulation, surgical education

## Abstract

**Purpose:**

Surgical simulation of microvascular anastomosis has become increasingly popular. There are several living and silicone models available. Current silicone models fail to accurately reproduce a vessel’s loose adventitial layer, which may lead to the development of improper microsurgical technique. Our purpose is to create a realistic 3-dimensional microsurgical simulator that incorporates an adventitial vessel layer for higher fidelity manipulation of vessels.

**Methods:**

A microvascular anastomosis simulator was manufactured using metal moulds and inorganic materials. Synthetic tubing was created with a metal cylinder, 1.65 mm in diameter, painted with 2 sequential layers of silicon with a shore hardness of 2A. Silicone was allowed to fully cure in-between layers. Vessel adventitia was created with a 100-micron polyester mesh adhered to the silicone vessel exterior. Once dry, the synthetic tube is removed from the metal cylinder is then clipped to reveal the inner lumen. Both Resident and attending physicians evaluated the model with and without the adventitial layer and completed a questionnaire.

**Results:**

Grasping and manipulation of the vessel were scored on Average score 4.5 and 3 out of 5, with adventitia and without, respectively (*P* = .00906). Usefulness as a teaching tool was scored on average 4.9 and 4.2, with adventitia and without, respectively (*P* = .0232). The analysis included: simulation realism, educational utility, and overall satisfaction. Responses in all domains were favourable, suggesting the utility of this model.

**Conclusion:**

We created a realistic, high fidelity microvascular anastomosis simulator that is low cost and easily reproducible. Initial feedback is encouraging regarding realism, educational utility, and overall usefulness. Further validation is required to assess its effectiveness in resident education and skill transfer to the operating room.

## Abstract (fr)

**Objectif:**

La simulation chirurgicale de l’anastomose microvasculaire gagne en popularité. Il existe plusieurs modèles de simulation vivants ou en silicone. Les modèles actuels en silicone ne réussissent pas à reproduire la couche adventitielle lâche, ce qui peut entraîner une technique microchirurgicale inappropriée. Les chercheurs voulaient créer un simulateur microchirurgical tridimensionnel réaliste doté d’une couche adventitielle pour manipuler les vaisseaux avec plus de fiabilité.

**Méthodologie:**

Les chercheurs ont fabriqué un simulateur d’anastomose microvasculaire au moyen de moules métalliques et de matières inorganiques. Ils ont créé des tubulures synthétiques à l’aide d’un cylindre métallique d’un diamètre de 1,65 mm, qu’ils ont peint de deux couches séquentielles de silicone d’une dureté Shore A de 2. Ils ont laissé le silicone durcir complètement entre les couches et ont créé la couche adventitielle à l’aide d’une maille de polyester de 100 microns fixée à l’extérieur du vaisseau de silicone. Une fois sèche, la tubulure synthétique est retirée du cylindre métallique, puis coupée pour révéler la lumière interne. Des résidents et des médecins traitants ont évalué le modèle avec et sans la couche adventitielle et rempli un questionnaire.

**Résultats:**

La saisie et la manipulation du vaisseau ont obtenu un score moyen de 4,5 et de 3 sur 5, avec et sans la couche adventitielle, respectivement (*p* = 0,00906). L’utilité de ce vaisseau comme outil d’enseignement a obtenu un score moyen de 4,9 et de 4,2, avec et sans la couche adventitielle, respectivement (*p* = 0,0232). L’analyse incluait le réalisme de la simulation, l’utilité pour l’enseignement et la satisfaction globale. Les réponses étaient favorables dans tous les domaines, ce qui laisse croire à l’utilité du modèle.

**Conclusion:**

Les chercheurs ont créé un simulateur d’anastomose microvasculaire haute-fidélité réaliste, à la fois peu coûteux et facile à reproduire. Les premiers commentaires sont encourageants pour ce qui est du réalisme, de l’utilité pour l’enseignement et de l’utilité globale. Son efficacité lors de l’enseignement aux résidents et du transfert du savoir en salle d’opération devra être validée davantage.

## Introduction

As part of any plastic surgery training program, *microsurgery* is a core pillar among the fund of knowledge that must be learned and applied. Access to the information behind the theory of microsurgery is in abundance. Access to clinical application of these theories has become increasingly more challenging, that is the combination of an ever-reducing allocation of time in workplace training with a highly demanding skill that has high stakes in the case of error has led to the establishment of simulation as an integral part of microsurgical training.^1,2^ Surgical simulators aim to improve operative skills and patients’ safety by allowing trainees to recreate tasks modelled before and after surgical procedures. Benefits of surgical simulation include reduced time spent in the operating room teaching basics, maximizing the benefit from actual cases, ensuring adequate case volume, skill transfer from the simulator to the operating room, and improved patient outcomes.^3,4^ Microsurgical simulation has become increasingly popular among training programs for these reasons.

Currently, there are several living and non-living commercial microsurgical simulation models available. Rat vessels, chicken thigh, plastic and latex tubes, are among the most common simulators.^5-7^ The living simulators implicate large costs for few models. Loh et al survey all living microsurgical models, they state that despite the well-documented efficacy of animal models on the acquisition of surgical skills, several ethical, financial, and accessibility issues exist.^8,9^ In a recent systematic review performed by Abi-Rafeh et al non-living microsurgical simulators are highlighted to play a promising role in the future of microsurgical training for the repetitive motions associated with surgery and circumvent many of the issues associated with animal models.^9^ Other studies have shown that silicone tubing can yield comparable results to that of animal models with retention of skills at 4 months.^9^

Our Authors hypothesize that non-living synthetic tubes do not incorporate an adventitial layer to the microvessel. Subsequentially, this requires the operator to grasp the vessel wall in order to anastomose the vessel, leading to the practice and acquisition of poor microsurgical technique. Our purpose is to create a realistic 3-dimensional microsurgical simulator that incorporates an adventitial vessel layer for higher fidelity manipulation of vessels. Secondary objectives include preliminary subjective testing by residents and microsurgery staff.

## Materials and Methods

The synthetic tubing was created with 1.65 mm diameter metal stent coated with 2 sequential layers of soft silicone with a Shore hardness of 2A. The shore hardness is a scale uses to measure the consistency of elastomers such as silicone. The number ranges from 0 to 100 and measuring rubbers soft to ridged, respectively. The “A” Scale gradings apply to silicones that range from very flexible to semi-rigid, and “D” scale gradings are assigned to more rigid plastics.^10^ A non-stick spray was applied to the metal cylinder to facilitate removal. The silicone was allowed to fully cure in-between layers. Vessel adventitia was created with a 100-micron polyester mesh adhered to the silicone vessel exterior.

The resident and attending physicians evaluated the model with and without the adventitial layer and completed a semi-structured questionnaire. There was no time allotted to practice with the models before evaluation. Each evaluator spent approximately 15 minutes evaluating both models. Five-point Likert Scale questions were used to evaluate both variations of the model (Table 1). General comments and open-ended feedback were recorded. Pre-rating questions screened for Level of Training, Number of Anastomosis performed, and comfort with performing Anastomosis. A 9-0 ethilon sutures were used to perform the anastomosis.

**Table 1.** Resident Survey Responses Evaluating the Models Based on Realism, Educational Utility, and Overall Usefulness.^a^

	Resident physicians without adventitia (score out of 5)	Resident physicians with adventitia (score out of 5)
Simulation realism		
Model is Anatomically accurate	3.6	4
Tissue feel is realistic	3.7	4.1
Tissue elasticity realistic	4	4.1
Ability to grasp vessel with forceps is realistic	3.3	4.4
Resistance of suture through vessel is realistic	3.9	4.3
Educational utility		
Useful for teaching microvascular anastomosis	4.3	4.9
Useful for improving operative technique	4.1	4.9
Overall usefulness		
I would recommend this model to other trainees	3.7	5
This model should be incorporated into our training curriculum	3.4	5

^a^ Responses were graded on a 5-point Likert scale: 5, strongly agree; 4, agree; 3, neutral; 2, disagree; and 1, strongly disagree.

A novel microsurgical model was created that incorporated an adventitial layer. The material cost to produce each vessel was less than $1. Seven residents (ranging from postgraduate year 1 [PGY1] to PGY5) most of whom have previous microsurgery experience and 3 microsurgery staff compared the models. A comparison was made between models using a Mann-Whitney *U* Test with *P* < .05.

Institutional Ethics Board review was deemed unnecessary. This study was unfunded.

## Results

Grasping and manipulation of the vessel were scored on Average score 4.5 and 3 out of 5, with adventitia and without, respectively (*P* = .00906). Usefulness as a teaching tool was scored on average 4.9 and 4.2, with adventitia and without, respectively (*P* = .0232).

Three staff evaluated both models. All found the adventitia resulted in a more realistic grasp and improved fidelity over the non-adventitia model. All staff agreed that the model with adventitia should be integrated into the training program over the non-adventitia model. Both Staff and Resident evaluations were measured through subjective assessment.

## Discussion

**Figure 1.**
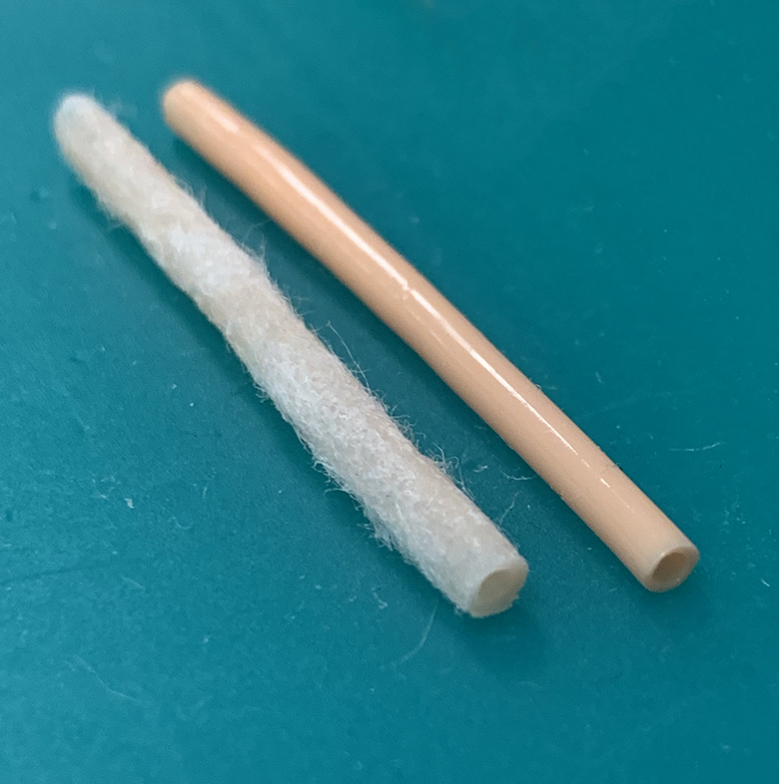
Microsurgical simulator with adventitial layer (left), and without adventitial layer (right).

Living animal models, such as rat femoral and aortic vessels offer a higher fidelity at a higher cost. Residents may not always have access to a laboratory for use of these models. Non-living animal models offer higher fidelity at a lower cost, disadvantages to these models are they tend to expire or perish and there are issues with contamination, commonly requiring a separate operation space due to concerns of contamination and sterility. Synthetic models are the least ideal for fidelity, with the potential for the acquisition of poor microsurgical skills. However, they are lower cost and offer enhanced convenience. Our study was interested in closing the gap between the medium to low fidelity models (Figure 1).

A limitation to the use of our synthetic tubing simulator, in contrast to an “in vivo” model such as the rat femoral anastomosis, is that there is no blood flow through it. Managing flow and recognizing anastomotic leaks is a crucial step in learning microsurgery. We also recognize that animal models do offer a higher fidelity of tissue manipulation and acquisition of microsurgical skills. Research comparing our model with adventitia and without, to performance on living models would further our understanding of the fidelity of our model. Also, the assessment model we used is a subjective evaluation and had not been previously validated.

Recognizing that each model has its place in resident education, low fidelity models useful for basic components such as microscope setup, handling of instruments and suture, and knot tying. High fidelity for more subtle technical components such as tissue dissection and manipulation, atraumatic technique, as well as suture placement and tensioning.

Both models appear useful for microsurgery training and the addition of adventitia increases the fidelity and is not cost-prohibitive. Multiple comments from senior residents and staff surgeons requesting the ability to clean the adventitia to practice preparing the vessel; this is not currently possible but being investigated. This novel model likely narrows the fidelity gap between synthetic and animal models while offering enhanced convenience.

## Conclusion

We created a realistic 3-dimensional microsurgical simulator that incorporates an adventitial vessel layer for higher fidelity manipulation of vessels that is low cost and easily reproducible. Initial feedback is encouraging regarding realism, and educational utility. Further investigation into other vessel properties is currently ongoing.

## Supplementary Material

Supplementary material
